# Supplementary material for: The role of molecular xenomonitoring for assessing the impact of Ivermectin, Diethylcarbamazine Citrate and Albendazole triple-drug treatment against lymphatic filariasis: A cross-sectional study in the Kenyan coastal region
Source: PLOS Glob Public Health. 2025 Dec 16;5(12):e0004877. doi: 10.1371/journal.pgph.0004877 (PMC12707650; doi:10.1371/journal.pgph.0004877)
Supplement: Attachment — Submitted filename: PLOS Iver Meben.docx [file pgph.0004877.s001.docx]

**Full Title:**

“The role of molecular xenomonitoring in the assessment of the impact of Ivermectin, Diethylcarbamazine Citrate and Albendazole triple-drug therapy treatment to the lymphatic filariasis infection levels in the Kenyan coast.”

**Comment:** The title initially gives an impression that this is an interventional study or an evaluation of an interventional study like a clinical trial. I understand this is an observation study, the title should also give that impression to the reader. The title is comprehensive but slightly long and contains redundancy (“triple-drug” “therapy” and “treatment"). It also doesn’t clearly communicate the study design. Consider streamlining it. For instance: “Impact of Ivermectin, Diethylcarbamazine Citrate and Albendazole Therapy on Lymphatic Filariasis in Coastal Kenya: A Longitudinal Assessment Using Molecular Xenomonitoring.” The question is what is your primary objective? The role of MX or the Impact of Ivr, DCC, and Alb. The short title is in order.

**Abstract**

**Line 22-23:** “The triple-drug therapy of Ivermectin, Diethylcarbamazine (DEC), and Albendazole (IDA)...”

**Comment:** Putting the DEC abbreviation and then IDA makes it confusing on what is what. No need for abbreviating DEC at this point.

**Line 29-31:** “Traps used included CDC light, Gravid, and BioGents® (BG) traps at baseline, and only CDC light and Gravid traps at IIS-1.”

**Comment:** Was the change in trapping methods between surveys by design or was this unintentional, if the latter could be a potential limitation.

**Line 34-38:** “At baseline, 37,732 mosquitoes were collected: 0.07% Anopheles, 2.3% Aedes, and 97.6% Culex. Of 486 pools tested, 30 (0.26%) were positive for W. bancrofti DNA. At IIS-1, 71,634 mosquitoes were collected: 0.46% Anopheles and 99.5% Culex. Of 1,695 pools tested, 22 (0.06%) were positive. Significant reductions were observed in filarial DNA in mosquitoes (76.9%, p < 0.001) and human antigenemia (52.1%, p = 0.004) after two treatment rounds.”

**Comment:** This is a bit confusing. The number of positive pools is 30, but the percentage (30/486)*100 = 6.17%. The 0.26% figure appears to be from somewhere else, but not clear where. Similarly, (22/1695)*100 = 1.30%.

**Introduction**

**Line 63-68:** “Lymphatic filariasis (LF) is a vector-borne parasitic disease belonging to a category of diseases known as Neglected Tropical Diseases (NTDs). The disease is caused by three filarial worms: namely, Wuchereria bancrofti, Brugia malayi and B. timori. The filarial parasites are transmitted by different types of mosquitoes, which vary from one geographical region to another. In Kenya, the infection is known to be transmitted by mosquitoes belonging to two genera: Anopheles (Anopheles funestus and Anopheles gambiae) and Culex (Culex quinquefasciatus).”

**Comment:** This is all assumed to be common knowledge and no need for references?

**Line 73-78:** “The World Health Organization (WHO) established the Global Programme for Elimination Lymphatic Filariasis (GPELF) in the year 2000 in response to the World Health Assembly resolution WHA50.29 formulated in 1997. The resolution urged Member States to immediately initiate activities to eliminate LF as a public health problem by the year 2020. The GPELF has the mandate to steer efforts toward the interruption of LF transmission, and morbidity management and disability prevention.”

**Comment:** Add reference.

**Methods**

Line 131-137; “The study received ethical approval from the Scientific and Ethics Review Unit (SERU) of Kenya Medical Research Institute (KEMRI) (KEMRI/SERU protocol No. 4523). Permission for human and mosquito sample collection was further sought and obtained from the respective county governments. Community mobilization and sensitization was done through meetings organized by the local leaders at the village/cluster level. The study participants and household heads provided a written informed consent for sample collection which included that further molecular analysis would be conducted on the samples.”

**Comments:** While the ethics statement is strong and shows how thoroughly the authors addressed the ethics of the study from National up to the individual level. However, clarity is required for the statement of whom a written consent was gotten from “study participants and household heads.” For clarity and to emphasize the principle of individual autonomy, state that individual written informed consent was obtained from all competent adult participants, and permission was also sought from household heads in accordance with cultural norms. This avoids any ambiguity about whether a household head's consent was considered sufficient for all members.

If children (<12 years) or adolescents under the age of 18 (12 to 17 years) were included as participants, the statement should explicitly describe the process for obtaining assent in addition to consent.

The phrase “further molecular analysis” is good. But what of storage of samples this is not explicitly stated, and for how long? Or samples were not stored, and only analysis was done?

Example of a suggested revision “The study received ethical approval from the Scientific and Ethics Review Unit (SERU) of the Kenya Medical Research Institute (KEMRI) (KEMRI/SERU protocol No. 4523). Permission to conduct the study and collect human and mosquito samples was further obtained from the respective county governments. Community mobilization and sensitization were conducted through meetings with local leaders and community members at the village level. All adult participants provided individual written informed consent. For participants under 18, written parental permission was obtained, along with written assent from the minor where appropriate. The consent process, conducted in the local language, included provisions for non-literate participants and specified that samples could be stored for future molecular analysis related to vector-borne disease research.”

**Line 139-142:** “This study comprised of repeated cross-sectional entomological surveys that were carried out in the two evaluation units (EU), namely Lamu County and Jomvu sub-County in Mombasa County. In each EU, mosquito samples were collected from 30 randomly and 5 - 9 purposively selected villages also referred to as clusters at baseline and at IIS-1.”

**Comment:** The statement “repeated cross-sectional entomological surveys” doesn’t clearly explain the study design. Does repeated cross-sectional mean before and after study? Is this a longitudinal design? Or two separate unrelated cross-sectional surveys combined during analysis? The mixed sampling strategy (“random” and “purposive”) is confusing. How was this done? The rationale for the purposive selection should be clearly explained.

**Line 144-150****:** “The mosquito samples were collected in the same sites as the population-based surveys as described by Njenga et al(20). Lamu East and Lamu West sub-Counties situated in Lamu County were considered to be a single EU due to their low population while Jomvu sub-County in Mombasa County was the second EU. Lamu County consists mainly of the mainland and several islands in the Indian Ocean that spread to the Northern part of the Kenyan coast and border Somalia. Jomvu sub-County is located to the west of Mombasa city and characterized by urban, peri-urban and rural areas.”

**Comment:** Mentioning “The mosquito samples were collected in the same sites as the population-based surveys as described by Njenga et al(20).” is good but not informative of the study site. Mention that the study sites are two (Lamu East, Lamu West, and Jomvu sub-Counties) and mosquito samples were collected as they were in the survey by Njenga et al before going into the explanation. The reader would have to pick this information later which would be confusing when one doesn’t know if it is two or three sites from the beginning. One of the strengths of this study is it’s one health approach (human and vectors), it would be great to also add the environmental component. How is the weather? This would be important for the reader. I am assuming the climate affects the population of these mosquitos and by extension the disease itself throughout the year. The text describes the sites but does not explicitly state “why” these specific sites were chosen for the study. Adding a brief justification would strengthen the methods section. Were they selected based on a known high burden of the disease being studied, specific ecological characteristics, or to represent contrasting settings (e.g. island/mainland vs. urban/rural)?

In the first sentence, ensure there is a space before the citation and a period after "al.", for example: “...as described by Njenga et al. (20).”

In the description of Lamu County, consider replacing “spread to” with a more formal term like “extending to” or “which extend to”.

The study setting was not explicitly stated. Still not clear when, where, and why the IDA therapy was administered to the population, and by who.

I have not seen a section on the study population, some information on this was embedded in other sections. Study population for humans, and for mosquitoes is important. Also, explicitly state the inclusion and exclusion criteria.

**Line 153-154:** “...children from 5 - 9 years and an older population (age ≥ 10 years in baseline and age ≥ 18 years at IIS-1).”

**Comment:** The change in the age bracket for the older population between baseline and follow-up is a notable difference. Please provide a brief justification for this change. Also provide the age range, ≥ 18 years is not specific. This part should be under the subheading study population. Mention the inclusion and exclusion criteria.

**Line 156-158**: “In the field, consent for the enrolment of all the participants was done on an opt-in basis and further, only children whose parents and/or legal guardians gave informed consent were recruited.”

**Commen**t: This should be covered in the ethics section to make it more compressive and complete. I expected to see this in that section.

**Line 156-158**: “...Mf circulation…”

**Commen**t: Check if “Mf” is the standard abbreviation for microfilariae

**Line 176-177**: “...only CDC light and Gravid traps were used [at IIS-1].”

**Comment:** The Methods section should explicitly state why the “BG-sentinel trap” was dropped. Was it due to low yield of relevant vectors, logistical reasons, or another factor? This is mentioned in the discussion but should be briefly stated here for methodological completeness.

**Results**

**Line 261-262:** “During the baseline survey, mosquitoes were collected in 210 households spread across the 35 villages in each EU…. During IIS-1, the collection was done in two rounds to increase the number of Anopheles genus. In each round, 750 households were sampled across 74 clusters (39 in Jomvu and 36 in Lamu)….”

**Comments**: This information should have been mentioned in the methods not appearing for the first time in the results section.

**Line 281-354:**

**Comment:** Tables 2 to 4 are descriptive but implied to be inferential with prevalence, 95% confidence interval, and relative reduction (RR) reported. The reader would expect the authors to present results on measures of impact in addition to descriptive results based on the title. These measures include; The Relative Risk (RR), Risk Difference (Attributable Risk), Absolute Risk Reduction (ARR), Population Attributable Risk, and Attributable Fraction are all measures used in epidemiology to quantify the effect of an exposure (like a risk factor or treatment) on the occurrence of a health outcome.

First, the structure of the tables need to be refined for clarity. The n reported is confusing with the number of pools.
