# Supplementary material for: The role of molecular xenomonitoring for assessing the impact of Ivermectin, Diethylcarbamazine Citrate and Albendazole triple-drug treatment against lymphatic filariasis: A cross-sectional study in the Kenyan coastal region
Source: PLOS Glob Public Health. 2025 Dec 16;5(12):e0004877. doi: 10.1371/journal.pgph.0004877 (PMC12707650; doi:10.1371/journal.pgph.0004877)
Supplement: Attachment — Submitted filename: Rebuttal letter.docx [file pgph.0004877.s003.docx]

**Review comments**

**Iver Meben comments**

**Full Title:**

“The role of molecular xenomonitoring in the assessment of the impact of Ivermectin, Diethylcarbamazine Citrate and Albendazole triple-drug therapy treatment to the lymphatic filariasis infection levels in the Kenyan coast.”

**Comment:** The title initially gives an impression that this is an interventional study or an evaluation of an interventional study like a clinical trial. I understand this is an observation study, the title should also give that impression to the reader. The title is comprehensive but slightly long and contains redundancy (“triple-drug” “therapy” and “treatment"). It also doesn’t clearly communicate the study design. Consider streamlining it. For instance: “Impact of Ivermectin, Diethylcarbamazine Citrate and Albendazole Therapy on Lymphatic Filariasis in Coastal Kenya: A Longitudinal Assessment Using Molecular Xenomonitoring.” The question is what is your primary objective? The role of MX or the Impact of I vr, DCC, and Alb. The short title is in order.

**Response 1: We thank the reviewer for the insightful suggestion. The title has been revised to clearly reflect the study design and the redundancy removed.**

**Abstract**

**Line 22-23:** “The triple-drug therapy of Ivermectin, Diethylcarbamazine (DEC), and Albendazole (IDA)...”

**Comment:** Putting the DEC abbreviation and then IDA makes it confusing on what is what. No need for abbreviating DEC at this point.

**Response: The abbreviation DEC in line 22 has been removed to avoid any form of confusion and defined in line 93**

**Line 29-31:** “Traps used included CDC light, Gravid, and BioGents® (BG) traps at baseline, and only CDC light and Gravid traps at IIS-1.”

**Comment:** Was the change in trapping methods between surveys by design or was this unintentional, if the latter could be a potential limitation.

**Response: The BioGents® (BG) trap was dropped because results of the baseline survey supported the existing literature that *Aedes* mosquitoes are not primary LF vectors in Kenya. This information is contained in line 201 – 203**

**Line 34-38:** “At baseline, 37,732 mosquitoes were collected: 0.07% Anopheles, 2.3% Aedes, and 97.6% Culex. Of 486 pools tested, 30 (0.26%) were positive for W. bancrofti DNA. At IIS-1, 71,634 mosquitoes were collected: 0.46% Anopheles and 99.5% Culex. Of 1,695 pools tested, 22 (0.06%) were positive. Significant reductions were observed in filarial DNA in mosquitoes (76.9%, p < 0.001) and human antigenemia (52.1%, p = 0.004) after two treatment rounds.”

**Comment:** This is a bit confusing. The number of positive pools is 30, but the percentage (30/486)*100 = 6.17%. The 0.26% figure appears to be from somewhere else, but not clear where. Similarly, (22/1695)*100 = 1.30%.

**Response: Thank you for this comment. As indicated in the paper, the samples were analysed in pools of up to 25 mosquitoes. Therefore, pools had varying number of mosquitoes and thus the pools had different statistical powers. Thus, the prevalence was not determined through the standard method of using the number of positive pools divided by total number of analysed pools multiplied by a 100. Instead, the prevalence was computed using a software package PoolTools v0.1.4, where the pools were entered individually into the software and the respective number of mosquitoes per pool. This information is provided in line 250 – 257.**

**Introduction**

**Line 63-68:** “Lymphatic filariasis (LF) is a vector-borne parasitic disease belonging to a category of diseases known as Neglected Tropical Diseases (NTDs). The disease is caused by three filarial worms: namely, Wuchereria bancrofti, Brugia malayi and B. timori. The filarial parasites are transmitted by different types of mosquitoes, which vary from one geographical region to another. In Kenya, the infection is known to be transmitted by mosquitoes belonging to two genera: Anopheles (Anopheles funestus and Anopheles gambiae) and Culex (Culex quinquefasciatus).”

**Comment:** This is all assumed to be common knowledge and no need for references?

**Thanks. A reference (1) has been added in line 68.**

**Line 73-78:** “The World Health Organization (WHO) established the Global Programme for Elimination Lymphatic Filariasis (GPELF) in the year 2000 in response to the World Health Assembly resolution WHA50.29 formulated in 1997. The resolution urged Member States to immediately initiate activities to eliminate LF as a public health problem by the year 2020. The GPELF has the mandate to steer efforts toward the interruption of LF transmission, and morbidity management and disability prevention.”

**Comment:** Add reference.

**Thanks. A reference (7) has been added in line 77.**

**Methods**

Line 131-137; “The study received ethical approval from the Scientific and Ethics Review Unit (SERU) of Kenya Medical Research Institute (KEMRI) (KEMRI/SERU protocol No. 4523). Permission for human and mosquito sample collection was further sought and obtained from the respective county governments. Community mobilization and sensitization was done through meetings organized by the local leaders at the village/cluster level. The study participants and household heads provided a written informed consent for sample collection which included that further molecular analysis would be conducted on the samples.”

**Comments:** While the ethics statement is strong and shows how thoroughly the authors addressed the ethics of the study from National up to the individual level. However, clarity is required for the statement of whom a written consent was gotten from “study participants and household heads.” For clarity and to emphasize the principle of individual autonomy, state that individual written informed consent was obtained from all competent adult participants, and permission was also sought from household heads in accordance with cultural norms. This avoids any ambiguity about whether a household head's consent was considered sufficient for all members.

If children (<12 years) or adolescents under the age of 18 (12 to 17 years) were included as participants, the statement should explicitly describe the process for obtaining assent in addition to consent.

The phrase “further molecular analysis” is good. But what of storage of samples this is not explicitly stated, and for how long? Or samples were not stored, and only analysis was done?

Example of a suggested revision “The study received ethical approval from the Scientific and Ethics Review Unit (SERU) of the Kenya Medical Research Institute (KEMRI) (KEMRI/SERU protocol No. 4523). Permission to conduct the study and collect human and mosquito samples was further obtained from the respective county governments. Community mobilization and sensitization were conducted through meetings with local leaders and community members at the village level. All adult participants provided individual written informed consent. For participants under 18, written parental permission was obtained, along with written assent from the minor where appropriate. The consent process, conducted in the local language, included provisions for non-literate participants and specified that samples could be stored for future molecular analysis related to vector-borne disease research.”

**Response: The consenting was done during the field evaluations and the procedures/details of participants consenting were captured under a related publication by Njenga *et al.,* 2024 that reported details of participant recruitment, sample collection, field testing and results. However, having carefully considered your comments, we have added the details of the ethical consideration in general in line 130 - 138.**

**For storage – the informed consent was sought to store samples but the timelines for storage were not explicit. Our plans were to store the samples temporarily awaiting acquisition of the relevant reagents which usually takes time due to importation logistics. The current study did not have a plan for long term storage of the samples.**

**Line 139-142:** “This study comprised of repeated cross-sectional entomological surveys that were carried out in the two evaluation units (EU), namely Lamu County and Jomvu sub-County in Mombasa County. In each EU, mosquito samples were collected from 30 randomly and 5 - 9 purposively selected villages also referred to as clusters at baseline and at IIS-1.”

**Comment:** The statement “repeated cross-sectional entomological surveys” doesn’t clearly explain the study design. Does repeated cross-sectional mean before and after study? Is this a longitudinal design? Or two separate unrelated cross-sectional surveys combined during analysis? The mixed sampling strategy (“random” and “purposive”) is confusing. How was this done? The rationale for the purposive selection should be clearly explained.

**Response: As it indicated in this comment, this study analysed samples from two separate cross-sectional surveys. First survey was a baseline before treatment and second (cross-sectional) survey was after two rounds of treatment. Purposive clusters were clusters that were selected intentionally based on the LF prevalence from the existing data and they were selected in consultation with local health authorities while random clusters were selected purely by chance from the list of all villages in the area. The intentions of this categorizing the clusters were for the study to provide information on what would be the most ideal cluster type (in Monitoring & Evaluation guidelines) for evaluating impact of the new treatment in future. These details are found in line 143 - 146**

**Line 144-150****:** “The mosquito samples were collected in the same sites as the population-based surveys as described by Njenga et al(20). Lamu East and Lamu West sub-Counties situated in Lamu County were considered to be a single EU due to their low population while Jomvu sub-County in Mombasa County was the second EU. Lamu County consists mainly of the mainland and several islands in the Indian Ocean that spread to the Northern part of the Kenyan coast and border Somalia. Jomvu sub-County is located to the west of Mombasa city and characterized by urban, peri-urban and rural areas.”

**Comment:** Mentioning “The mosquito samples were collected in the same sites as the population-based surveys as described by Njenga et al(20).” is good but not informative of the study site. Mention that the study sites are two (Lamu East, Lamu West, and Jomvu sub-Counties) and mosquito samples were collected as they were in the survey by Njenga et al before going into the explanation. The reader would have to pick this information later which would be confusing when one doesn’t know if it is two or three sites from the beginning. One of the strengths of this study is it’s one health approach (human and vectors), it would be great to also add the environmental component. How is the weather? This would be important for the reader. I am assuming the climate affects the population of these mosquitos and by extension the disease itself throughout the year. The text describes the sites but does not explicitly state “why” these specific sites were chosen for the study. Adding a brief justification would strengthen the methods section. Were they selected based on a known high burden of the disease being studied, specific ecological characteristics, or to represent contrasting settings (e.g. island/mainland vs. urban/rural)?

**Response: We appreciate this observation. As explained earlier, this study targeted laboratory analysis of samples collected at baseline and during a cross-sectional survey after two rounds of IDA MDA. As field procedures and data are reported by Njenga *et al*., 2024, we focused on providing a reference with those details including description of the study sites. The current study delves much on the laboratory analysis but provide the key highlights of two mother studies as a way of giving background of the origin of the samples.**

In the first sentence, ensure there is a space before the citation and a period after "al.", for example: “...as described by Njenga et al. (20).” **Response: Done Line 149**

In the description of Lamu County, consider replacing “spread to” with a more formal term like “extending to” or “which extend to”. **Response: Edited accordingly in line 152**

The study setting was not explicitly stated. Still not clear when, where, and why the IDA therapy was administered to the population, and by who. **Response: Thank you for above comment. Below I am providing the clarification as follows: -**

**In 2017, WHO approved the use of the new Ivermectin, Diethylcarbamazine Citrate and Albendazole (IDA) triple drug therapy drug for the treatment of lymphatic filariasis. This was done after findings of previous small-scale studies showed that the combination of the 3 drugs (triple therapy) can clear LF infection faster and its safe for human use. Kenya was chosen to be one of the countries where the drug was to be tried for large-scale administration. Additionally, during the large-scale administration trial, Kenyan would be used to develop guidelines for monitoring and evaluation framework for the new drug therapy. Therefore, two studies were conceived. The first study also known as the baseline survey (3721), was to conduct field testing and collect both human dried blood spot (DBS) & mosquito samples (for further analysis) before administration of the IDA drug. This was done from the 14^th^ October – 18^th^ November 2018 under the approval KEMRI/SERU protocol No. 3721. Thereafter, two rounds of IDA drug were administered in Nov/Dec 2018 and Nov 2019. The second study was done from the 24^th^ November 2020 - 18^th^ July 2021. This study (4099) also referred to as IDA Impact Survey 1 (IIS-1) similarly conducted the field testing and collected human DBS & mosquito samples for further analysis too. This was done under approval KEMRI/SERU protocol No. 4099. These two studies had two arms; human and vector arm as indicated above. In the human arm, LF testing in the field was done using filarial test strip (FTS). A dried blood spot (DBS) sample was also collected during field LF testing from each participant for further antibody analysis in the laboratory. For the FTS positive persons, night blood sample was collected between 10.00pm – midnight (time when microfilariae can be found in peripheral blood) for microfilariae (Mf) testing using microscopy technique. Additionally, a dried blood spot was collected from the FTS positive persons during the collection of a night blood sample. This second DBS sample was to be used for LF testing using molecular-based PCR technique. In the vector arm, there was no any testing in the field but instead mosquito samples (LF vector) were collected in the same villages/clusters as human samples. In the field, mosquito samples were sorted by genus, packaged and transported to KEMRI, Nairobi. These mosquito and night blood DBS samples were analysed in the KEMRI labs. The data reported in this paper is mainly from laboratory based molecular (PCR) analysis of the mosquito and night DBS samples that were collected during the two main studies (baseline & IIS-1) indicated above. However, the findings of this PCR assays were compared with field-based findings (FTS & Mf) to establish whether the two sets of results can provide similar message on status of LF infection. The lab analysis reported in the current study was approved under KEMRI/SERU protocol No. 4523 as indicated in line 150 of the manuscript**

**We also provide the rationale behind selection of the two sites in line 96 - 99**

I have not seen a section on the study population, some information on this was embedded in other sections. Study population for humans, and for mosquitoes is important. Also, explicitly state the inclusion and exclusion criteria.

**Response: We appreciate the observation. As explained earlier, this study targeted laboratory analysis of samples collected in two previous cross-sectional surveys. The human survey field procedures and data are reported by Njenga et al., 2024. However, we have added a section on Study populations below the community human surveys in line 172 - 180. It would be good to note that the primary objective of the current study was to analyse filarial DNA in mosquito vectors and thus human survey was not the main analysis. However, at some points we also compared the data with human indicators of infection as way of establishing whether they would convey similar message.**

**Line 153-154:** “...children from 5 - 9 years and an older population (age ≥ 10 years in baseline and age ≥ 18 years at IIS-1).”

**Comment:** The change in the age bracket for the older population between baseline and follow-up is a notable difference. Please provide a brief justification for this change. Also provide the age range, ≥ 18 years is not specific. This part should be under the subheading study population. Mention the inclusion and exclusion criteria.

**Response: Thank you for the observation. The upper age group was changed to ≥18 years for the impact assessment because it became clear that the 10–17 years age group would be challenging for NTD programs to assess and interpret if it were to become global guidance, due to varying rates of boarding school attendance and the age at which children enter the work sector. The sample size of the 5–9 years-old children that was required for the survey in each area was selected to be similar to the sample sizes recommended for the WHO-recommended TAS survey. This was reported in Njenga et al., 2024. We have provided these details in line 174 - 180**

**Line 156-158**: “In the field, consent for the enrolment of all the participants was done on an opt-in basis and further, only children whose parents and/or legal guardians gave informed consent were recruited.”

**Commen**t: This should be covered in the ethics section to make it more compressive and complete. I expected to see this in that section.

**Response: This was deliberately put here because it was relevant to human sampling in the field. However, we have added the necessary ethical and additional details in line 135 - 138**

**Line 156-158**: “...Mf circulation…”

**Commen**t: Check if “Mf” is the standard abbreviation for microfilariae

**Response: This is a good observation. Some authors use “Mf” whereas others use “mf”; we preferred to use “Mf”.**

**Line 176-177**: “...only CDC light and Gravid traps were used [at IIS-1].”

**Comment:** The Methods section should explicitly state why the “BG-sentinel trap” was dropped. Was it due to low yield of relevant vectors, logistical reasons, or another factor? This is mentioned in the discussion but should be briefly stated here for methodological completeness.

**Response: The BioGents® (BG) trap was dropped because results of the baseline supported the existing literature that Aedes mosquitoes are not primary LF vectors in Kenya. This information is contained in line 201 – 206.**

**Results**

**Line 261-262:** “During the baseline survey, mosquitoes were collected in 210 households spread across the 35 villages in each EU…. During IIS-1, the collection was done in two rounds to increase the number of Anopheles genus. In each round, 750 households were sampled across 74 clusters (39 in Jomvu and 36 in Lamu)”

**Comments**: This information should have been mentioned in the methods not appearing for the first time in the results section.

**Response: Thank you for the observation. The details have now been included into the Mosquito sampling and pooling section in line 183 - 186**

**Line 281-354:**

**Comment:** Tables 2 to 4 are descriptive but implied to be inferential with prevalence, 95% confidence interval, and relative reduction (RR) reported. The reader would expect the authors to present results on measures of impact in addition to descriptive results based on the title. These measures include; The Relative Risk (RR), Risk Difference (Attributable Risk), Absolute Risk Reduction (ARR), Population Attributable Risk, and Attributable Fraction are all measures used in epidemiology to quantify the effect of an exposure (like a risk factor or treatment) on the occurrence of a health outcome.

First, the structure of the tables need to be refined for clarity. The n reported is confusing with the number of pools.

**Response: Tables 2, 3 and 4 show infection prevalence during baseline survey and impact survey (i.e. survey done after two rounds of treatment). Usually, if the prevalence at impact survey is significantly lower than prevalence at baseline survey then it indicates that the IDA (the intervention) had an impact in reducing the infection prevalence. In these tables therefore, we showed the prevalence at each survey time point and assessed whether the reduction in prevalence was significant using relative reduction technique (i.e. a technique that assesses and quantifies the reduction in the prevalences as well as their significance). The statistical description of relative reduction technique has been provided in the data management and analysis section line 253 – 257. The relative reduction takes a form of multivariable mixed effect model.**

**Reviewer 1**

**GENERAL:**

There are a few minor typographical issues (e.g., inconsistent spacing before and after punctuation and citations) that should be addressed during final proofreading. Still, they do not detract from the overall clarity of the manuscript. I recommend a thorough grammar and style review to ensure the manuscript meets publication standards.

**Response: Done within the text**

**RESULT:**

"during baseline assessment" is repeated in lines 265–266.

**Response: edited accordingly. Line 269**

The dominance of the Culex genus is restated multiple times in similar ways; it can be made more concise.

**Response: Thanks for the comment. The edits were done within the text**

Use consistent formatting for genus names (italicize Anopheles, Aedes, Culex).

**Response: Thanks for the comment. The edits were done within the text**

It’s unclear what "IIS-1" refers to for readers who haven’t seen the acronym defined recently. Consider writing it out or reminding the reader.

**Response: We appreciate your comment. We have doubled checked that we defined the term IIS-1 on first use so that the reader can refer to it if need be. Line 28**

The tables are generally informative and support the results well. However, a few tables appear overly condensed or too small, making the data difficult to read or interpret clearly. Consider increasing font size and spacing for proper reading. If feasible, split very dense tables into multiple simpler ones.

**Response: Thank you comment. We are of a considered opinion that at the publication stage all the tables will be properly formatted and their fonts standardized for easy reading.**

**Reviewer** **2**:

This manuscript presents timely and important research evaluating the impact of triple-drug IDA mass drug administration on lymphatic filariasis (LF) using molecular xenomonitoring (MX) in coastal Kenya. The study offers valuable insights that are highly relevant for LF elimination programs and supports the operational value of MX as a surveillance tool. The integration of entomological data with human infection prevalence strengthens the quality of the findings.

**Strengths:**

The study design, combining human and vector-based data, is innovative and fits well within global efforts to enhance NTD surveillance.

The laboratory protocols, including pooling strategies and qPCR methods, are clearly described and adhere to high scientific standards.

Ethical approvals and informed consent procedures are adequately documented.

The manuscript demonstrates a commitment to open data, in line with PLOS policies.

**Response: Thank you for the above complements, we feel very encouraged.**

**Major Areas for Improvement:**

**Title Simplification**

The current title is excessively long and includes redundant phrasing ("therapy treatment"). Consider shortening to enhance clarity and reader engagement.

**Response: Thank you for this comment. We have revised the title.**

**Sampling Strategy and Household Coverage**

While the study mentions the use of both purposive and random villages, it does not explain the rationale for these selections. Please clarify why these methods were used and their implications for the representativeness of the findings.

There is no information on household coverage; please specify how many households were sampled at each site and whether the same ones were revisited in follow-up surveys.

**Response: The details of clusters selection are provided in line 143 - 146. This selection was meant to establish whether the two types of villages would yield similar results and for possible inclusion/adoption into the guidelines for the IDA monitoring and evaluation. Number of households indicated in line 183 and line 185.**

**Statistical Reporting**

Some of the reported prevalence changes lack p-values or confidence intervals. Please provide statistical tests for key comparisons to support the strength of your conclusions.

**Response: All the reduction values together with p-values are provided in their respective tables except relative increase. The statistical description of relative reduction technique has been described in the data management and analysis section line 253 – 257.**

Explain clearly how percentage reductions (e.g., “76.9% decrease”) were calculated and whether they are statistically significant.

**Response: The statistical description of relative reduction technique has been described in the data management and analysis section line 253 – 257.**

**Programmatic Relevance**

The discussion could be expanded to provide practical guidance for national LF programs:

**Response: Thank you for the insights. Our discussion is targeted to the impact of IDA and use of filarial DNA as proxy for the level of infection during monitoring and evaluation. At the end, we provide conclusions for consideration by the LF programs and WHO.**

How can MX be integrated into routine surveillance?

**Response: This study actually recognizes the place for integrating MX with surveillance of the other mosquito borne diseases in one health approach in line 495 - 500.**

What are the training, cost, and scalability considerations?

**Response: This is a very good idea. However, our study did not collect data on the costs. This would probably be a rich ground for further research as per our recommendation in line 501 - 502**

How could MX be leveraged for integrated vector-borne disease surveillance under a One Health approach?

**Response: This is possible and in line with our observations/recommendation in line 497 - 500**

**Minor Issues to Address:**

Ensure scientific names (e.g., Wuchereria bancrofti) are consistently italicized.

**Response: Edited in the text accordingly**

Expand figure legends so that each is interpretable independently of the text.

**Response: Thank you for the observation. We find the figure legend submitted separately to be descriptive enough. The figure captions are also descriptive enough.**

Some sentences (e.g., Lines 411–414) are overly long or awkward; consider editing for clarity and fluency.

**Response: Thank for pointing out this edit, we have rephrased the statement accordingly in line 414 – 416**

**Conclusion:**

This is a well-executed and timely study that makes a significant contribution to the literature on LF elimination. I recommend major revisions to clarify the sampling strategy, reinforce statistical reporting, and expand the discussion of programmatic applications. With these refinements, the manuscript will be a strong fit for PLOS Global Public Health

**Reviewer 3**

This study provides some insight into the impact of a triple drug combination therapy on lymphatic filariasis infection in Kenya. Grammar and phrasing needs to be reviewed in the editing phases. The writing can be more concise and the authors should consider removing words such as "probably", "not necessarily" and other adverbials.

**Thank for pointing out this edit, we have addressed the specified words in line 414 – 416 and others across the entire paper.**

**INTRODUCTION:**

The author immediately introduces Kenya and then discusses the Pacific endemic islands. It is not clear why. Where is FL most prevalent? How many people are affected? How big is the problem in Kenya?

**Response: Thank you for the comments. We have removed the statement on the Pacific endemic islands. The information about LF in Kenya is provided in line 68 – 71 and 83-91**

In lines 97-101, the two study sites are first mentioned. Is there a reason why these study sites were chosen? Is there any reference to support this? Are lines 99-101 meant to describe the purpose of the current study?

**Response: Thank you for the comment. We provide the reference of the status of LF that largely guided the site selection. Site selection was also based on the published data in the provided reference and unpublished information obtained from the LF program and local authorities. This is provided in line 89 – 91.**

**The purpose of the current is provided in closing paragraph of the Introduction chapter in line 118 - 122**

**METHODS:**

The manuscript does not have a detailed purpose and explanation of the statistical methods used in the methods section. The spatial differences (random vs. purposive) and temporal differences (baseline vs. IIS-1) could be made much clearer. It was unclear to me why there were "random" and "purposive" clusters, the meaning of these groups were not outlined in the methods. Please outline these clearly for the reader in the methods section. Aside from this, I can see that the statistics used were relevant to the nature of the work.

**Thank you for the complement. We have added details on the analysis plan. For clarity our comparison was between the baseline survey and impact survey that was done after two rounds of MDAs (Intervention) with IDA. This can be found in line 249 - 257**

**Purposive clusters were clusters that were selected intentionally based on the LF prevalence from the existing data and selected in consultation with local health authorities while random were clusters that were selected purely by chance from the list of all villages in the area. The intention of this categorization was to provide information on what would be the most ideal cluster type for evaluating impact of the new treatment in case it is adopted for use by the other endemic countries. The clarification is provided under study design in line 143 - 146**

**RESULTS**

Line 268 describes “the collection”. Can the author elaborate on what this refers to: the collection of mosquito samples or human related samples?

**Response: Clarified to mean collection of mosquitoes in line 271**

Section 3.1 is said to describe the demographic characteristics; however it outlines the sample collection for the mosquitoes. Consider rephrasing the title for this section.

**Response: Thank for the comment. The section has been rephrased to – General characteristics in line 263.**

Table 2 shows a very low prevalence of LF. I am uncertain how prevalence was calculated here.

E.g. For “all sites” in Jomvu at baseline: If number of positive pools / number of pools analysed = 30 / 323 = 0.0929. Where does 0.40 (0.27-0.56) come from?

**Response: The samples were analysed in pools of up to 25 mosquitoes. Therefore, each pool had varying numbers of mosquitoes and thus the power of each pool was deemed not to be the same. Thus, the prevalence was not determined through the standard method of using the number of positive pools divided by total number of analysed pools multiplied by a 100. Instead, the prevalence was computed using a software package PoolTools v0.1.4, where the pools were entered individually into the software and the respective number of mosquitoes per pool. This information is provided in line 250 - 257**

For table 2, Consider adding that the samples were obtained from mosquitoes.

**Response: Done added “in mosquitoes” in line 309**

As the tables go on, I am still uncertain about the calculations of prevalence. Please specify how these were calculated.

**Response: The samples were analysed in pools of up to 25 mosquitoes. Therefore, each pool had varying numbers of mosquitoes and thus the power of each pool was deemed not to be the same. Thus, the prevalence was not determined through the standard method of using the number of positive pools divided by total number of analysed pools multiplied by a 100. Instead, the prevalence was computed using a software package PoolTools v0.1.4, where the pools were entered individually into the software and the respective number of mosquitoes per pool. This information is provided in line 250 - 257**

**DISCUSSION**

It would be more impactful if the first paragraph (lines 368-381 only discussed the novel findings from this paper, before going into how this paper relates to the remainder of the literature.

Lines 476-479 discuss how the study sites allowed for generalisable conclusions to be made. This is a bold point given that there are many epidemiologies associated with disease status. Please elaborate on this by providing more context on these study sites in the methods and how they are similar epidemiologically to other settings where LF is prevalent.

The conclusion "This study demonstrates that LF transmission has likely been interrupted in Lamu County and Jomvu sub-county, Mombasa County in the Kenyan Coast thus warranting stoppage of the MDA." Is

**Response: Thank you for the insight. It is our considered opinion that the study explores and compare the findings with other studies. Further, it provides the key findings, recommendations for consideration in policy formulation and on the areas for further research.**

**Reviewer 4**

Thank you for giving chance to review this very impactful and deserved work. The data availability found supporting the conclusions and the research thesis incorporated research ethical principles. The recommendation and conclusion was relevant and draw based the authors finding.

**Response: Thank you for the complements**

**Reviewer 5**

Thank you for the opportunity to review your manuscript, “The role of molecular xenomonitoring in the assessment of the impact of Ivermectin, Diethylcarbamazine Citrate and Albendazole triple-drug therapy treatment to the lymphatic filariasis infection levels in the Kenyan coast.” This is an important study that provides valuable data on the impact of IDA therapy and the utility of molecular xenomonitoring in the Kenyan context. The work has clear programmatic relevance. To improve the manuscript for potential publication, I offer the following comments for your consideration.

**Major Comments**

1. Methodological Clarity and Study Design

The Methods section requires significant revision to provide the clarity and detail necessary for readers to fully understand and evaluate the study.

**Response: We have enhanced methods section as provided in the Track changed document. We have used the comments from other reviewer to provide clarifications and additional details. Each review is provided and line numbers indicated accordingly**

Study Design (Line 139): The description “repeated cross-sectional entomological surveys” is ambiguous. Please clarify if this was a longitudinal study with a pre- and post-intervention assessment of the same areas, or two independent cross-sectional surveys. A clear, standard term for the study design should be used consistently.

**Response: As it indicated in this comment, this study analysed samples from two separate cross-sectional surveys. First survey was before treatment and second survey was after two rounds of treatment. Purposive clusters were clusters that were selected intentionally based on the LF prevalence from the existing data and they were selected in consultation with local health authorities while random clusters were selected purely by chance from the list of all villages in the area. The intentions of this categorizing the clusters were for the study to provide information on what would be the most ideal cluster type (in Monitoring & Evaluation guidelines) for evaluating impact of the new treatment in case its adopted for use by other endemic countries. Line 143 - 146**

Sampling Strategy (Line 141): The use of a mixed sampling strategy (“random” and “purposive”) needs justification. Please explain the rationale for selecting some villages purposively and describe the criteria used for this selection.

**Purposive clusters were clusters that were selected intentionally based on the LF prevalence from the existing data and they were selected in consultation with local health authorities while random clusters were selected purely by chance from the list of all villages in the area. The intentions of this categorizing the clusters were for the study to provide information on what would be the most ideal cluster type (in Monitoring & Evaluation guidelines) for evaluating impact of the new treatment in case its adopted for use by other endemic countries. Line 143 - 146**

Study Population: The manuscript lacks a dedicated "Study Population" section. Information is currently scattered. Please create a distinct section that clearly defines the human and mosquito populations studied, including explicit inclusion and exclusion criteria for both. Details on household numbers (lines 261-262) should be moved from the Results to this new section in the Methods.

**Thank you for the comment. We have introduced a separate section of study – 2.5 Study population. However, this section is relatively summarized for two main reasons: -**

1. **This study report on the analysis of the sample collected in other studies and thus brief highlights of the studies is deemed sufficient**
2. **The current study provides a reference of the results of the two mother studies and thus this area is sufficiently covered under the said reference.**

**Separately, we have also provided details on household numbers have been added in lines 183 & 185**

Study Setting (Line 144-150): Please provide more context.

**Response. We have additional details and provided reference that will have more details on study site in line 148 - 154.**

Justify why these specific sites were chosen (e.g., known high LF burden, representing diverse ecological settings, etc.). **Additional context for the sites and reasons for the choice details are provided in lines 68 – 71, 88 – 91 and 96 -99**

Provide essential details about the IDA mass drug administration (MDA) itself: When, where, and by whom was the therapy administered? This context is crucial for understanding the intervention whose impact is being assessed.

**Response: Thank you for above comment. As provided by our response to a similar clarification that was sought by another reviewer, I am providing the clarification again as follows: -**

**In 2017, WHO approved the use of the new Ivermectin, Diethylcarbamazine Citrate and Albendazole (IDA) triple drug therapy drug for the treatment of lymphatic filariasis. This was done after findings of previous small-scale studies showed that the combination of the 3 drugs (triple therapy) can clear LF infection faster and its safe for human use. Kenya was chosen to be one of the countries where the drug was to be tried for large-scale administration. Additionally, during the large-scale administration trial, Kenyan would be used to develop guidelines for monitoring and evaluation framework for the new drug therapy. Therefore, two studies were conceived. The first study also known as the baseline survey (3721), was to conduct field testing and collect both human dried blood spot (DBS) & mosquito samples (for further analysis) before administration of the IDA drug. This was done from the 14^th^ October – 18^th^ November 2018 under the approval KEMRI/SERU protocol No. 3721. Thereafter, two rounds of IDA drug were administered in Nov/Dec 2018 and Nov 2019. The second study was done from the 24^th^ November 2020 - 18^th^ July 2021. This study (4099) also referred to as IDA Impact Survey 1 (IIS-1) similarly conducted the field testing and collected human DBS & mosquito samples for further analysis too. This was done under approval KEMRI/SERU protocol No. 4099. These two studies had two arms; human and vector arm as indicated above. In the human arm, LF testing in the field was done using filarial test strip (FTS). A dried blood spot (DBS) sample was also collected during field LF testing from each participant for further antibody analysis in the laboratory. For the FTS positive persons, night blood sample was collected between 10.00pm – midnight (time when microfilariae can be found in peripheral blood) for microfilariae (Mf) testing using microscopy technique. Additionally, a dried blood spot was collected from the FTS positive persons during the collection of a night blood sample. This second DBS sample was to be used for LF testing using molecular-based PCR technique. In the vector arm, there was no any testing in the field but instead mosquito samples (LF vector) were collected in the same villages/clusters as human samples. In the field, mosquito samples were sorted by genus, packaged and transported to KEMRI, Nairobi. These mosquito and night blood DBS samples were analysed in the KEMRI labs. The data reported in this paper is mainly from laboratory based molecular (PCR) analysis of the mosquito and night DBS samples that were collected during the two main studies (baseline & IIS-1) indicated above. However, the findings of this PCR assays were compared with field-based findings (FTS & Mf) to establish whether the two sets of results can provide similar message on status of LF infection. The lab analysis reported in the current study was approved under KEMRI/SERU protocol No. 4523 as indicated in line 130 - 131 of the manuscript**

Considering the influence of climate on vector populations, adding brief details on the local environmental conditions (e.g., rainy/dry seasons) would enrich the site description.

**We have provided a reference with in depth description of the sites in line 148 – 154 and a reference with further description of the study sites. In the said reference the details of field procedures are also included while the current study provided highlight of the site and details of the field tests. The current study was mainly geared towards downstream laboratory analysis of the samples.**

Changes in Methodology: The manuscript notes several changes between the baseline and follow-up surveys. These must be justified within the Methods section.

Age Brackets (Line 153): Explain the reason for changing the age criteria for the older population from ≥10 years at baseline to ≥18 years at IIS-1. Please also provide a specific upper age limit or range for the ≥18 group.

**Response: The upper age group was changed to ≥18 years for the impact assessment because it became clear that the 10–17 years age group would be challenging for NTD programs to assess and interpret if it were to become global guidance, due to varying rates of boarding school attendance and the age at which children enter the work sector. The sample size of the 5–9 years-old children that was required for the survey in each area was selected to be similar to the sample sizes recommended for the WHO-recommended TAS survey. This was reported in Njenga et al. 2024. The details have also been included in the methods under a newly included study population section in lines 172 - 180**

Mosquito Traps (Line 176): State in the Methods why the BG-sentinel trap was dropped for the IIS-1 survey.

**Response: The response is provided elsewhere as follows: - The BioGents® (BG) trap was dropped because results of the baseline supported the existing literature that Aedes mosquitoes are not primary LF vectors in Kenya. This information is contained in the 201 – 203.**

2. Ethical Considerations (Lines 131-137)

While the ethics statement is strong, it can be enhanced for greater clarity and completeness.

Consent Process: The phrase “study participants and household heads provided a written informed consent” could be ambiguous. To emphasize individual autonomy, please clarify that individual written informed consent was obtained from all competent adult participants, and that permission from household heads was sought in line with cultural norms, not as a substitute for individual consent.

Assent for Minors: If children or adolescents under 18 were included, the process for obtaining their assent, in addition to parental/guardian consent, must be explicitly described.

Sample Storage: The consent for “further molecular analysis” is noted, but please specify if this included consent for long-term storage of samples and for what duration.

Suggested Revision: Please consider incorporating elements from the following example to improve clarity: “The study received ethical approval from the Scientific and Ethics Review Unit (SERU) of the Kenya Medical Research Institute (KEMRI) (KEMRI/SERU protocol No. 4523). Permission to conduct the study and collect human and mosquito samples was further obtained from the respective county governments. Community mobilization and sensitization were conducted through meetings with local leaders and community members at the village level. All adult participants provided individual written informed consent. For participants under 18, written parental permission was obtained, along with written assent from the minor where appropriate. The consent process, conducted in the local language, included provisions for non-literate participants and specified that samples could be stored for future molecular analysis related to vector-borne disease research.”

**Response: Thank you for seeking the clarification. Response is provided to a similar question raised by another reviewer as follows: -**

**The nature of the current study was that there was no direct contact between participants and our study. Therefore, most of the ethical consideration that government interaction during sample collection and their use downstream were handled. The current study recognizes this ethical consideration and was additionally approved to use the said samples in compliance with the tenets of the ethical approval in the two mother studies. However, we have added more details of the ethical consideration as suggested in the comments in line 134 - 138.**

**For storage – the informed consent was sought to store samples but the timelines for storage were not explicit. Our plans were to store the samples temporarily awaiting acquisition of the relevant reagents which usually takes time due to importation logistic. The current study did not have a plan for long term storage of the samples.**

3. **Reporting and Analysis of Results**

Pool Positivity vs. Estimated Prevalence (Abstract, Lines 34-38): There is a significant discrepancy between the reported percentages (0.26% and 0.06%) and the raw pool positivity rates that can be calculated from the data provided (30/486 = 6.17% and 22/1695 = 1.30%). Please clarify in the text that the reported percentages are the estimated DNA prevalence from PoolTools and are distinct from the raw pool positivity rate. This is a critical point for reader comprehension.

**Response:** **The samples were analysed in pools of up to 25 mosquitoes. Therefore, each pool had varying numbers of mosquitoes and thus the power of each pool was deemed not to be the same. Thus, the prevalence was not determined through the standard method of using the number of positive pools divided by total number of analysed pools multiplied by a 100. Instead, the prevalence was computed using a software package PoolTools v0.1.4, where the pools were entered individually into the software and the respective number of mosquitoes per pool. This information is provided in line 250 - 257**

Measures of Impact (Tables 2-4): The study title implies an assessment of "impact." While the descriptive results are valuable, the analysis would be strengthened by reporting formal measures of impact beyond relative reduction. Please consider including measures such as Absolute Risk Reduction (ARR) or Risk Difference to provide a more complete picture of the intervention's effect.

**Response: Tables 2, 3 and 4 show infection prevalence during baseline survey and impact survey (i.e. survey done after two rounds of treatment). Usually, if the prevalence at impact survey is significantly lower than prevalence at baseline survey then it indicates that the IDA (the intervention) had an impact in reducing the infection prevalence. In these tables therefore, we showed the prevalence at each survey time point and assessed whether the reduction in prevalence was significant using relative reduction technique (i.e. a technique that assesses and quantifies the reduction in the prevalence as well as their significance). The statistical description of relative reduction technique has been provided in the data management and analysis section line 253 – 257. The relative reduction takes a form of multivariable mixed effect model.**

Table Structure: The structure of the tables, particularly the reporting of 'n', can be confusing when trying to distinguish between the number of mosquitoes and the number of pools. Please refine the table legends and column headers for clarity.

**Response: Thank you for highlighting this important labelling oversight. We have labelled the tables accordingly and defined “n” in the legend of the respective tables.**

**Minor Comments**

Title: The current title is long, contains some redundancy ("therapy treatment"), and could give the impression of a clinical trial. Please consider revising it to better reflect the observational study design and primary objective. For instance: “Impact of Ivermectin, Diethylcarbamazine Citrate and Albendazole Therapy on Lymphatic Filariasis in Coastal Kenya: A Longitudinal Assessment Using Molecular Xenomonitoring.”

**The title has also been edited in line with the comments by the reviewers by removing the word “therapy” and rephrased to remove redundancy – Line 2**

Abstract (Line 22): Defining Diethylcarbamazine as "(DEC)" immediately before defining the three-drug combination as "(IDA)" is confusing. Since DEC is not referred to again on its own, consider removing this abbreviation.

**Response: The abbreviation DEC in line 22 has been removed to avoid any form of confusion and defined in line 93**

Introduction (Lines 63-78): Several foundational statements about LF and the WHO's GPELF are presented without citations. While some may be considered common knowledge, it is best practice to reference key facts and programmatic statements. Please add appropriate references.

**Response: Thank you for the comment. The references (1) and (7) in lines 68 & 77 respectively have been added.**

Formatting (Line 144): Please ensure consistent formatting for citations (e.g., space before parenthesis, period after "al.").

**Response: This has been addressed accordingly in line 149**

Terminology (Line 158): Please confirm that "Mf" is the standard, accepted abbreviation for microfilariae in the context of this journal.

**Response: Response: This is a good observation. Some authors use “Mf” whereas others use “mf”; we preferred to use “Mf”.**

Thank you for your work on this important topic. I believe that addressing these points will significantly improve the manuscript's clarity, rigor, and impact. Please find the detailed line by line comments attached.
